# Top-down threat bias in pain perception is predicted by higher segregation between resting-state networks

**DOI:** 10.1162/netn_a_00328

**Published:** 2023-12-22

**Authors:** Veronika Pak, Javeria Ali Hashmi

**Affiliations:** Department of Neurology and Neurosurgery, McGill University, Montreal, QC, Canada; McConnell Brain Imaging Centre, Montreal Neurological Institute, Montreal, QC, Canada; Department of Anesthesia, Pain Management, and Perioperative Medicine, Nova Scotia Health Authority, Halifax, NS, Canada; Dalhousie University, Halifax, NS, Canada

**Keywords:** Pain perception, Bias, Top-down, Expectation, Prediction, Threat, Functional MRI, Functional connectivity, System segregation, Resting-state networks, Pain catastrophizing

## Abstract

Top-down processes such as expectations have a strong influence on pain perception. Predicted threat of impending pain can affect perceived pain even more than the actual intensity of a noxious event. This type of threat bias in pain perception is associated with fear of pain and low pain tolerance, and hence the extent of bias varies between individuals. Large-scale patterns of functional brain connectivity are important for integrating expectations with sensory data. Greater integration is necessary for sensory integration; therefore, here we investigate the association between system segregation and top-down threat bias in healthy individuals. We show that top-down threat bias is predicted by less functional connectivity between resting-state networks. This effect was significant at a wide range of network thresholds and specifically in predefined parcellations of resting-state networks. Greater system segregation in brain networks also predicted higher anxiety and pain catastrophizing. These findings highlight the role of integration in brain networks in mediating threat bias in pain perception.

## Author Summary

Some individuals are more sensitive to threats of receiving strong pain stimuli. In this study, we report that higher system segregation in brain networks is associated with a greater threat bias (a reliance on expected intensity of pain versus the actual sensory stimuli in the manipulated pain experiment). We verified the results with different predefined resting-state parcellations at different network sparsities, and also tested subject-driven parcellations. These findings demonstrate that higher integration between resting-state networks is conducive for integrating top-down expectation processes with changes in bottom-up noxious stimulus intensities. Network segregation in resting state is thus associated with poor sensory integration and also shows association with anxiety and fear of pain.

## INTRODUCTION

Any painful experience aids the learning of threat contingencies so that future experiences of pain can be inferred, and tissue damage prevented. These top-down processes, through which expectations influence pain, may rely on brain network architecture (Caras & Sanes, 2017; Choi et al., 2018). Parsing out individual differences in large-scale network features that govern pain perception and how expectations modulate pain intensity can be useful for understanding why people perceive pain differently and what goes awry in chronic pain conditions (Fields, 2018; Heathcote et al., 2020; Leeuw et al., 2007; Peters et al., 2002).

When a sensory experience relies more on prediction and less on sensory verification, the result is a biased perceptual experience that deviates from the objectively defined external cause. A key function of pain is to warn and direct attention towards threats (Kucyi & Davis, 2015; Legrain et al., 2011). Hence, in pain-averse individuals, expectations can have a strong hold on perception when noxious events are expected to be more intense (Aue & Okon-Singer, 2015; Bar-Haim et al., 2007; Kelley & Schmeichel, 2014; LeDoux, 2014; Smith et al., 2021). We have reported that a stepped increase in the threat of experiencing a strong pain-evoking stimulus can significantly magnify the pain even though the stimulus intensity was constant (Lim et al., 2020). Each cued increase in the expected stimulus strength linearly increased the perceived pain intensity (Kong et al., 2013; Lim et al., 2020). In some individuals, the increased threat of strong noxious stimuli produced increasingly more intense pain, and this variability was taken as an indicator of top-down threat bias (Aristi et al., 2022). Greater threat bias was predicted by higher pain-catastrophizing scores, low mindful awareness, and low pain tolerance (Lim et al., 2020), and was also associated with reduced integrity in white matter pathways needed in the brain for sensory and top-down integration (Aristi et al., 2022). Nevertheless, the effects of variability in large-scale functional connectivity patterns on pain perception remain unknown.

Brain networks consist of modules representing subnetworks where each may putatively serve distinct functions (Gozdas et al., 2019; Khan et al., 2022; Rubinov & Sporns, 2010; van den Heuvel & Sporns, 2013). These modules are in turn connected with each other through relatively sparse global connections (Baum et al., 2017). The balance of the number of connections within and between subnetworks, termed as system segregation/integration (SS), varies between individuals and is a useful feature that captures higher order cognitive brain functions (Cohen & D’Esposito, 2016; Misic & Sporns, 2016). It has been suggested that system integration in brain networks reflects cognitive efforts and has also been shown to predict performance accuracy in a working memory task (Cohen & D’Esposito, 2016). In contrast, greater clustering and more system segregation predicted the ability to adhere to prescribed training such as meditation, cognitive behavioral therapy, and exercise (Arnemann et al., 2015; Baniqued et al., 2018, 2019; Gallen et al., 2016; Saghayi et al., 2020). Although any specific function of network segregation is unclear, putatively, brain subnetworks that function relatively independently may have increased capacity for specialized functions but at the possible cost of a reduced capacity to integrate new information (Aristi et al., 2022; Bullmore & Sporns, 2012; Hashmi et al., 2017). For instance, higher clustering indices, which is a measure related to SS, has been reported to predict higher expectation effects towards the efficacy of a treatment (placebo analgesia; Hashmi et al., 2014). Note that when placebo analgesia and expectation effects are strong, they can be represented as a type of cognitive bias, where the error between actual and predicted sensation is not fully integrated and perception is more reliant on the top-down priors. This type of bias may be associated with individual differences in the capacity to integrate information from different subnetworks in the brain, but a direct mapping between top-down expectation effects and SS is yet to be clearly demonstrated.

Here we investigate top-down threat bias in healthy individuals at a wide range of network thresholds. We test reproducibility with three different a priori brain parcellations (i.e., previously predefined) and also with subject-specific parcellations. Results were verified by analyzing association with threat bias values as a continuous variable and also by grouping individuals into high- and low-bias groups. To further explore the role of SS in mediating group differences in threat bias, we studied the change in SS between rest and task. In addition, we tested the interrelationship between the brain measures and relevant variables including age, pain catastrophization, and anxiety.

## MATERIALS AND METHODS

### Participants

Forty-two healthy and right-handed individuals (21 female; ages 20–56 years; mean age ± *SD* of 31.23 ± 10.91 years) were recruited for the study and provided written informed consent. Exclusions criteria were as follows: (a) having concomitant acute or chronic pain; (b) taking medications for pain; (c) pregnancy; (d) history of cardiac, respiratory, or nervous system disease that could interfere with participation in the study or present potential for adverse outcome (e.g., asthma or psychiatric or mental disorders); or (e) contraindications to MRI scanning (e.g., cardiac pacemaker, metal implants [including titanium], dental braces, permanent retainers, or known fear of closed spaces). The study protocol was approved by the Nova Scotia Health Authority Research Ethics Board.

Of all 42 participants, 39 underwent structural T1-weighted MRI, diffusion-weighted imaging (DWI), functional MRI (fMRI) resting-state scan, and experimental pain task scans. The analysis of this study focuses on resting-state and experimental fMRI data collected from 39 participants. On structural analyses made on this dataset, see our previously reported findings (Aristi et al., 2022; Lim et al., 2020; Wang et al., 2022).

### Pain Task Experiment

The task was designed as a cognitive test of bias to evaluate the influence of top-down threat predictions on new sensory experience. Before proceeding to the MRI scanner, participants were informed that they would be shown information on a screen, experience thermal stimuli (heat), and be asked to rate the experienced pain intensity using a numeric scale.

**Figure 1.**
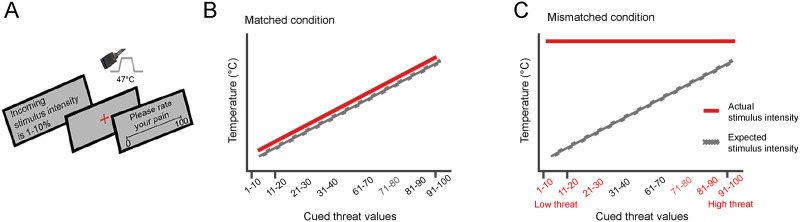
Experimental paradigm of the pain task performed by participants during fMRI acquisition. (A) In each trial, a threat cue stating the intensity of the incoming stimulus was shown on the screen. Heat pain stimulus was administered during the presentation of a red fixation cross on the screen. Then, participants were asked to rate their pain intensity on a scale of 0–100. (B) Matched condition: Threat cue predictions matched the heat stimuli intensity in a linear fashion, thus training participants to learn a linear schema on perceived pain, where actual stimulus intensity matched the expectations of the perceived pain. (C) Mismatched condition: In the last two runs (Tasks 2a and 2b), all threat cues were coupled with the maximum heat temperature of 47°C, as depicted on the plot. High threat cue values ranged from 70 to 98 and low threat cue values ranged from 8 to 32. The difference between pain ratings for high threat cues and low threat cues during the mismatched run was defined as top-down threat bias.

Each trial, threat cues on the screen would either predict the stimulus intensity in the form of a percentage range of 10 points (example: “the incoming heat stimulus is at 70%–80% intensity”) or prompt that the stimulus intensity is unknown (“the incoming heat stimulus intensity is unknown”). While participants were looking at a red fixation cross on the screen, the heat stimulation was delivered to their skin on their lower left leg (*tibialis anterior muscle*) via an ATS 30 × 30 mm thermode (PATHWAY system, Medoc). Participants were asked to rate their perceived pain intensity by using a 0–100 numerical rating scale (NRS; where 0 means no pain at all and 100 being the worst pain imaginable). Participants moved the cursor along the scale to select a number representing their pain level by pressing two buttons on an MRI-compatible response pad (Lumina LSC-400 controller, Cedrus). For a graphical representation of the task, see Figure 1A.

The procedure described above was repeated multiple times across four different runs including one matched-condition run, one level 1 mismatched-condition run, and two level 2 mismatched-condition runs. Mismatched runs were shortly named Task 1, Task 2a, and Task 2b, respectively.

In the matched run (see Figure 1B), threat cue values accurately predicted pain intensities in a linear fashion. Each 10-point increase in cued threat value was associated with a 0.4° increase in stimulus temperature (43.8–47°C temperature range). Therefore, during the matched condition, participants were taught to anticipate pain intensity based on the correctly predicted level of pain intensity.

In mismatched runs, threat cue values did not match the following heat stimuli, introducing the difference between expected and actual stimulus intensities. Three mismatched-condition runs were conducted to determine whether the linear relationship learned by participants during the matched condition carried over to subsequent runs of the task. During the level 1 mismatched run, all cue values ranging from 1% to 40% were coupled with the same low-heat 45°C stimulus, and cue values ranging from 60% to 100% were paired with a high-heat 47°C stimulus. There was no prediction error for cue ranges of 31%–40% and 91%–100%. In the remaining level 2 mismatched runs, all threat cue values were paired with the maximum temperature of 45°C or a 47°C. Participants’ reliance on the information presented by the visual cues, that is, their top-down bias, was measured using the trials in level 2 runs where the temperature was always 47°C (Figure 1C). The top-down threat bias was thus assessed by measuring the difference in pain ratings between the high and low threat cues, when the stimulus temperature remained the same for each participant.

### Top-Down Threat Bias Calculation

The effect of top-down threat cues on pain perception was calculated as a difference between averaged pain ratings in response to the low threat cue values (8%–32%) and to the maximum cue values (70%–98%). A greater difference indicated a stronger effect of threat cues on pain ratings. Only data collected during the mismatched level 2 runs, when the temperature remained at its peak (47°C) throughout the task, were used in these calculations.

We conducted a group comparison of individuals with different levels of threat bias to verify the results where threat bias was used as a continuous variable. Participants were assigned to the low threat bias (*n* = 15) and high threat bias (*n* = 17) groups by using a K-means clustering algorithm implemented in MATLAB (Lloyd, 1982). The algorithm assigned cluster memberships based on the data distribution itself and with minimal user decision-making. The high threat bias group included participants whose pain perception was more aligned with threat expectation and less with the actual heat stimulus. Participants who were being reclassified after each iteration (*n* = 7) and whose bias fell near the mean of the distribution were excluded from the group analysis.

### Psychological Measures

The pain catastrophizing scores and its three subscales (Rumination, Magnification, and Helplessness) were obtained using the Pain Catastrophizing Scale (PCS) questionnaire (Sullivan et al., 1995). State anxiety and trait anxiety were evaluated by self-reported State-Trait Anxiety Inventory (Spielberger et al., 1970).

### Data Acquisition

Structural and functional scans were collected using a 3T MRI scanner (Discovery MR750; General Electric Medical Systems, Waukesha, WI, USA) with a 32-channel head coil (MR Instruments, Inc.; Minneapolis, MN, USA). The experiment took place at the Halifax Infirmary Site, QEII Health Sciences Centre, Halifax, NS, Canada. Participants’ heads were fitted with foam padding to provide comfort and minimize motion, ear plugs were used to reduce sound levels, and reminders were given before each scan to keep their head still. Resting-state scan lasted 8 min. The following parameters were used for T1-weighted brain images (GE sequence 3D IR-FSPGR): field of view = 224 × 224 mm; in-plane resolution = 1 mm × 1 mm; slice thickness = 1.0 mm; TR/TE = 4.4/1.908 ms; flip angle = 9°. The fMRI BOLD (blood oxygenation level–dependent) sequence protocol used a multiband EPI sequence: field of view = 216 × 216 mm; in-plane resolution = 3 mm × 3 mm; slice thickness = 3.0 mm; TR/TE = 950/30 ms, SENSE factor of 2, acceleration factor of 3. There were 500 volumes for resting-state scans, 814 volumes for the training scan (not used in analysis), and 624 for the three pain task scans in total. For distortion correction, reverse phase-encoded images were also obtained for the application of FSL’s topup tool.

### Preprocessing of Resting-State Data

All functional datasets were corrected for field map–based distortion. Data were preprocessed with AFNI (https://afni.nimh.nih.gov/afni) and FSL (https://www.fmrib.ox.ac.uk/fsl), with the scripts provided by 1,000 Functional Connectomes Project (https://www.nitrc.org/projects/fcon_1000). All the following parameters for preprocessing were adapted from the parent study (Lim et al., 2020). Preprocessing with AFNI included (a) discarding the first five EPI volumes to allow signals to reach a steady state, (b) rigid-body motion correction of time series by aligning each volume to the mean image using Fourier interpolation, (c) skull stripping, and (d) getting an eighth image for use in registration. Preprocessing using FSL consisted of (e) spatial smoothing with a Gaussian kernel of full-width half-maximum = 6 mm, (f) grand-mean scaling of the voxel value, (g) bandpass temporal filtering between 0.005 and 0.3 Hz, (h) removal of linear and quadratic trends using Fourier transformation, and (i) mean-based intensity normalization.

The six motion parameters corresponding to rotational movement and cardinal displacement were generated using the FSL-based motion correction step in native functional space. In addition, two nuisance time courses were calculated for white matter and ventricles by using masks obtained from the image segmentation of the participant’s T1w data and applying a tissue-type probability threshold of 80%.

For analyzing data in standard space, FLIRT was used to perform registration of functional images to the MNI152 standard template (Fonov et al., 2011). This included (a) registration of native-space structural image to the MNI152 2-mm template using 12 *df* linear affine transformation, (b) registration of native-space functional image to high-resolution structural image with 6 *df* linear transformation, and (c) computation of native-functional-to-standard-structural warps using by concatenating matrices computed in steps (a) and (b).

For data quality verification, maximum framewise displacement (FD) and DVARS (difference of volume *N* to *N* + 1) were calculated to assess motion. For image quality criteria, participant data with maximum FD above 3 mm or DVARS outliers detected in more than 10% of the acquired data were removed from analysis. Mean FD was 0.12 (*SD* = 0.07) and mean DVARS was 46.9 (*SD* = 4.7). No images were excluded, as the highest maximum FD obtained and highest percentage of outliers in data detected for all participants was 2.65 mm and 8%. Maximum FD and maximum DVARS were used as nuisance covariates when correlating functional and behavioral metrics for further verification.

### Brain Parcellations

For each participant, image voxels were grouped into 131 regions of interest (ROIs), which were defined by the optimized Harvard-Oxford atlas for pain studies (Hashmi et al., 2014; Saghayi et al., 2020). BOLD time series were extracted from each voxel within each region and then averaged using FSL. The regions were grouped within the following five canonical resting-state networks (RSNs): subcortical, sensory, default mode, attention/executive, and language/memory (Hashmi et al., 2017).

Additional group-level parcellations with seven and 17 RSNs were employed from the open-source Yeo atlas (Yeo et al., 2011). The seven-network parcellation included 52 ROIs and the 17-network parcellation included 114 ROIs. The seven networks spanned visual, somatomotor, dorsal attention, ventral attention, limbic, frontoparietal, and default mode networks. The 17 networks presented a higher resolution extended parcellation with inclusion of sensory and motor cortices (Yeo et al., 2011). These estimated networks have been chosen for showing reliability across datasets and for being previously validated in region-based fMRI analyses (Yeo et al., 2011; Zhi et al., 2022).

### System Segregation Calculation

Whole-brain weighted system segregation was measured with a graph theory technique. To build the functional connectivity graph, the 131 × 131 adjacency matrix for each participant was created by calculating zero-lag Pearson’s linear correlations between the 131 BOLD time series. Negative correlation values and diagonal entries were removed from the correlation matrices. For selecting only significant functional connections unaffected by physiological or experimental noise, each matrix was thresholded and converted to a binarized adjacency matrix (van Wijk et al., 2010). Network thresholding was based on predefined correlation thresholds (percentage of the strongest connections) ranging from 0.20 to 0.50, with increments of 0.05. This range of possible thresholds was used to test for consistency of the results to account for spurious connections in unthresholded networks. Moreover, there is no consensus regarding an optimal threshold, and removing some connections may lead to the loss of important information at the cost of reduced sensitivity (Drakesmith et al., 2015; van den Heuvel et al., 2017; Zalesky et al., 2016). We used functions from the Brain Connectivity Toolbox and custom code implemented in MATLAB to quantify functional networks from the matrix (Avena-Koenigsberger et al., 2017; Rubinov & Sporns, 2010).

A measure for weighted system segregation for each network was defined as the difference between mean Fisher’s z-transformed within-network connectivity and mean Fisher’s z-transformed between-network connectivity; it was calculated by the following formula:

$$\mathrm{System}\;\mathrm{segregation}=\frac{{z^{-}}_{\text{w}}-{z^{-}}_{\text{b}}}{{z^{-}}_{\text{w}}},$$

where *z*^−^_w_ is the number of connections within a given network and *z*^−^_b_ is the number of connections between nodes (ROIs) within a given network and other networks, normalized by the total number of possible connections between networks. The code for the measure was employed from the study of Chan et al. (2014).

### Subject-Specific System Segregation Calculation

We defined subject-specific networks by adopting Girvan-Newman and Louvain algorithms, commonly used methods for community detection (Braga & Buckner, 2017; Harrison et al., 2015; Laumann et al., 2015; Yeo et al., 2011). This approach, known as modularity maximization process, aims to partition network’s nodes into brain modules (networks; Sporns & Betzel, 2016). Specifically, we calculated subject-specific weighted system segregation between a set of nonoverlapping modules (*m* as the total number of modules) calculated by the following formulas:

$$Q=\frac{1}{2m}\sum_{i,j}\left[A_{ij}-P_{ij}\right]\delta \left(c_{i},c_{j}\right),$$

where *Q* is a modularity coefficient, *A*_*ij*_ represents the number of connections between nodes *i* and *j*, *c*_*i*_ and *c*_*j*_ are the networks to which nodes *i* and *j* belong, *m* is the sum of all connectivity weights in the graph, *δ* is the Kronecker delta function, and *P*_*ij*_ stands for the expected number of connections according to a null model (Newman & Girvan, 2004). For an undirected network based on the Louvain method, the following formula was applied:

$$Q=\frac{1}{2m}\sum_{i,j}\left[A_{ij}-\frac{k_{i}k_{j}}{2m}\right]\delta \left(c_{i},c_{j}\right),$$

where *P*_*ij*_ = $\frac{k_{i}k_{j}}{2m}$, *k*_*i*_ and *k*_*j*_ are the sum of the connectivity weights attached to nodes *i* and *j*, and the delta-function *δ*(*c*_*i*_, *c*_*j*_) equals 1 if *i* = *j* and 0. Otherwise, 2*m* = ∑_*ij*_*A*_*ij*_ is the total number of connections in the network (Blondel et al., 2008).

## RESULTS

### Cue Threat Predictions Altered Pain Perception Evoked by a Heat Stimulus

**Figure 2.**
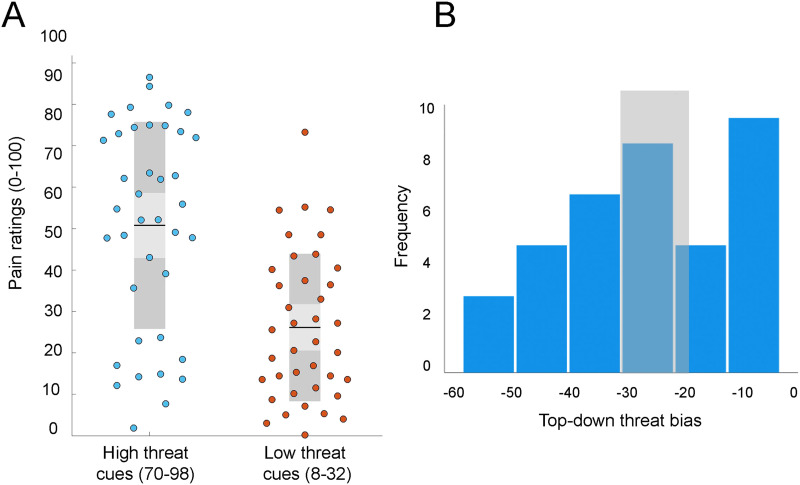
(A) Effects of high threat (70–98) and low threat (8–32) cue values on participants’ pain ratings during mismatched conditions. Pain ratings for high threat cues were significantly altered by threat bias formed by the linear schema. (B) Frequency distribution of perceptual threat bias scores calculated for healthy participants. The values for all participants were used as a continuous variable for testing associations with system segregation. For group analysis, participants lying on the left (*n* = 17) and right (*n* = 15) sides were classified by K-means clustering algorithms into the high threat bias and low threat bias groups, respectively. Shaded region shows the cutoff band of the K-means classification, where participants that lie within the band (*n* = 7) were excluded from the group analysis.

As we have previously reported, presenting threat predictions as cues prior to applying heat stimuli significantly influenced pain perception in healthy participants (Lim et al., 2020). Figure 2A demonstrates averaged pain ratings in response to low threat cues (8–32) and high-threat cues (70–98) averaged from three mismatched runs. These differences in pain ratings were related to the cues since in both conditions the stimuli were identical (peak stimuli intensity of 47°C), indicating the effect of linear schema on pain perception, that is, top-down threat bias. A greater difference in pain ratings was suggestive of a stronger top-down threat bias.

Figure 2B shows the distribution of bias scores calculated for 39 participants (−25.03 ± 15.86) as a difference in pain ratings in mismatched runs between low threat cues and high threat cues. The median of the distribution (−25) was within 10% of the mean, suggesting that the variability was normally distributed (*W* = 0.953, *p* = 103). K-means clustering was used to assign participants into two groups (high bias, *n* = 17; low bias, *n* = 15) based on their threat bias scores (high bias, −40.2 ± 8.31; low bias, −8.56 ± 5.4). The high bias group included individuals whose pain perception became intensified with increase in threat of a stronger stimulus. We have previously reported significant influence of all cue values on pain ratings in both threat groups (Aristi et al., 2022). Specifically, the increase in reported pain with increase in cued threat was more steep in the high bias group, while ratings in the low bias group showed less amplification with increase in cued threat (Aristi et al., 2022).

### System Segregation During Rest Predicts Top-Down Threat Bias

We first tested whether SS in five functional RSNs predicted top-down threat bias in 39 healthy individuals who underwent fMRI scans. We also compared differences in SS between individuals from the high bias and low bias groups. In addition we tested whether the findings reproduce when two other parcellations with different spatial resolutions from the Yeo atlas (Yeo et al., 2011) were used.

**Figure 3.**
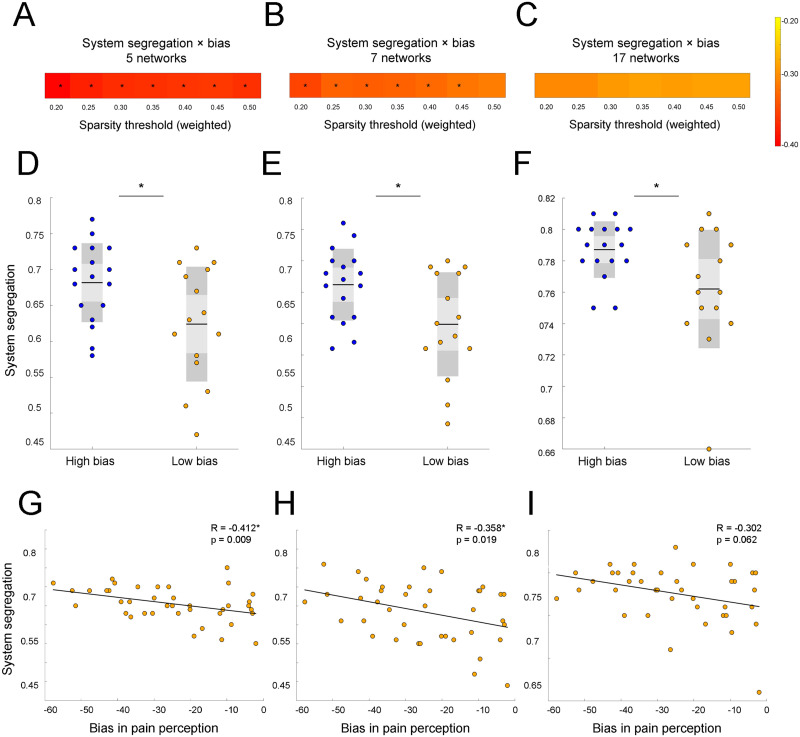
System segregation during resting state predicts top-down threat bias in pain perception at a wide range of network thresholds when tested with three different brain parcellations. (A) SS in five networks significantly correlates with threat bias at all sparsity thresholds (*p* < 0.05). (B) In seven networks, SS is associated with bias at almost all thresholds (*p* < 0.05). (C) A similar trend towards significance can be observed for correlations in 17 networks. The heat map on the right demonstrates the *R* values ranging from −0.2 (colored yellow) to −0.4 (red) for all parcellation schemes. (D–F) Participants in the high bias group demonstrated significantly higher SS compared with the low bias group at the 0.20 threshold (*p* = 0.026; *p* = 0.018; *p* = 0.03). Between-subject effect was significant for all parcellations (*p* < 0.05). (G) Scatterplot demonstrates a significant negative relationship between SS in five networks and threat bias at the 0.20 threshold (*p* = 0.009). (H) SS in seven networks also decreases with threat bias at the 0.20 threshold (*p* = 0.019) (I) Similar trend of a negative association between SS and threat bias can be observed in 17 networks at the 0.20 threshold. For each scatterplot, a line reflecting a linear regression between SS and threat bias in pain perception is depicted. Significant results are marked by asterisks (*).

As Figure 3 illustrates, higher weighted SS in five networks significantly correlated with higher top-down threat bias in pain perception at all seven sparsity thresholds (T^0.20–0.50^: *R* = −0.412, *p* = 0.009; *R* = −0.390, *p* = 0.014; *R* = −0.378, *p* = 0.018; *R* = −0.376, *p* = 0.018; *R* = −0.371, *p* = 0.02; *R* = −0.370, *p* = 0.02; *R* = −0.375, *p* = 0.019; Figure 3A). These findings were reproducible in seven networks: SS significantly correlated with threat bias at six out of seven sparsity thresholds (T^0.20–0.45^: *R* = −0.358, *p* = 0.025; *R* = −0.334, *p* = 0.038; *R* = −0.338, *p* = 0.035; *R* = −0.336, *p* = 0.037; *R* = −0.328, *p* = 0.041; *R* = −0.319, *p* = 0.048; Figure 3B). In comparison, the correlation analysis did not show significant correlations between threat bias and SS in 17 networks (*p* > 0.05). While results were not significant relative to the standard alpha level of 0.05, the *p* value was less than 0.10 at all sparsity thresholds. This result suggests that SS in 17 networks showed a trend similar to the findings from other parcellations and indicated that parcellations with lower spatial resolution were more effective in capturing the association between segregation and the threat bias (Figure 3C).

In group analysis, we found a significant between-subject effect (RM-ANOVA: *F*(1) = 4.816, *p* = 0.036) on SS in five canonical RSNs. This effect was reproducible in seven networks (RM-ANOVA: *F*(1) = 5.224, *p* = 0.030) and in 17 (RM-ANOVA: *F*(1) = 5.115, *p* = 0.031). Post hoc analysis showed that weighed SS in five networks was significantly different between the high threat bias and low threat bias groups at all seven sparsity thresholds (T^0.20–0.50^: *t* = −2.348, *p* = 0.026; *t* = −2.22, *p* = 0.034; *t* = −2.156, *p* = 0.039; *t* = −2.168, *p* = 0.038; *t* = −2.122, *p* = 0.042; *t* = −2.131, *p* = 0.041; *t* = −2.167, *p* = 0.038; independent *t* test, Figure 3D). Other parcellation networks produced similar results: the high bias group showed higher SS compared with the low bias group at all seven sparsity thresholds in seven networks (T^0.20–0.50^: *t* = −2.497, *p* = 0.018; *t* = −2.31, *p* = 0.028; *t* = −2.366, *p* = 0.025; *t* = −2.289, *p* = 0.029; *t* = −2.217, *p* = 0.034; *t* = −2.134, *p* = 0.041; *t* = −2.067, *p* = 0.047; independent *t* test, Figure 3E) and at all seven sparsity thresholds in 17 networks (T^0.20–0.50^: *t* = −2.284, *p* = 0.03; *t* = −2.337, *p* = 0.026; *t* = −2.257, *p* = 0.031; *t* = −2.235, *p* = 0.033; *t* = −2.263, *p* = 0.031; *t* = −2.226, *p* = 0.034; *t* = −2.196, *p* = 0.036; independent *t* test, Figure 3F). In the explorative analysis, we found that the high bias group showed more functional connectivity within RSNs. In comparison, the low bias group showed a larger number of significant connections between RSNs. These exploratory results illustrate that the high bias group shows greater SS relative to the low bias group (see the Supporting Information, Supplemental Figure 1).

### Subject-Specific Parcellations and System Segregation

Girman-Newman and Louvain algorithms for community detection were utilized to identify the number of network communities for each subject individually (Blondel et al., 2008; Newman & Girvan, 2004). Compared with the results from the predefined networks (optimized Harvard-Oxford and Yeo), weighted SS measured from subject-specific modules by the Girvan-Newman algorithm significantly correlated with top-down bias at only one sparsity threshold (T^0.20^: *R* = −0.337, *p* = 0.036). Correlations between subject-specific weighted SS based on the Louvain method were not significant at any of the sparsity thresholds.

### Changes in Segregation of Functional Brain Networks From Rest to Task

**Figure 4.**
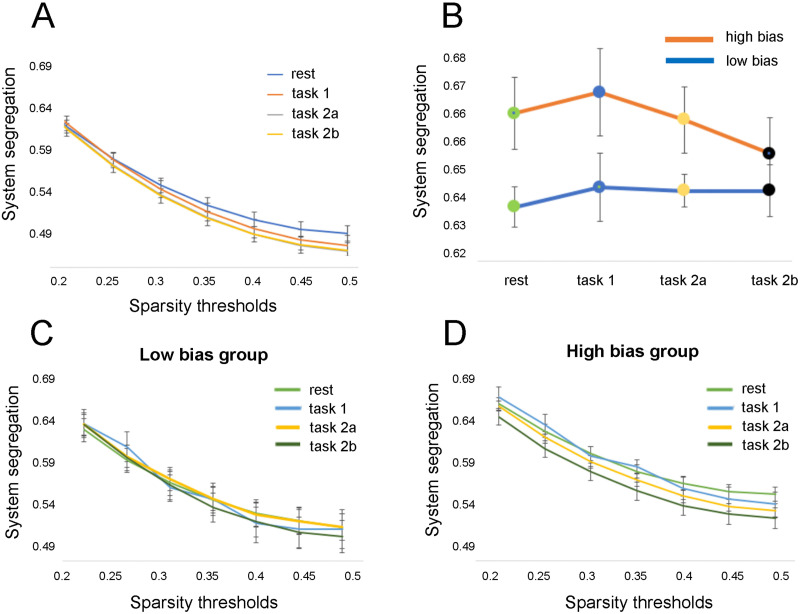
Participants in the high threat bias group showed significant changes in system segregation from rest to task scans compared with people in the low threat bias group. (A) Variability in SS values from rest to task showed significant interaction effect for type of scan and a sparsity threshold value (*p* < 0.01). (B) Participants in the high bias group showed significantly higher SS during rest, Task 1, and Task 2a compared with the low bias group at the 0.20 threshold (RM-ANOVA: *p* < 0.05). (C) No variation in SS from rest to task in the low bias group was identified across all sparsity thresholds (RM-ANOVA: *p* = 0.118). (D) Significant changes in SS between resting-state and task scans were observed in high bias group at different sparsity thresholds (RM-ANOVA: *p* < 0.01).

Figure 4 illustrates changes in SS values averaged across participants between resting-state scans and mismatched runs of the pain schema task. Two types of analyses were run. First, we looked at overall changes in SS with tasks; for that we looked at whether SS significantly changes with each subsequent scan. The RM-ANOVA revealed a significant overall effect on SS for all participants (*F*(18) = 4.968, *p* < 0.01 for type of scan × threshold). However, the between-subject effect was not significant (*F*(1) = 3.315, *p* = 0.079). The variability in SS between scans was not significant for the low bias group (*F*(27) = 1.347, *p* = 0.118 for type of scan × threshold; Figure 4C). Meanwhile, participants in the high bias group showed significant changes in SS from rest to task scans (type of scan × threshold, *F*(27) = 482.709, *p* < 0.01). The effect of the type of scan alone was also significant (*F*(3) = 478.572, *p* < 0.01). Figure 4D demonstrates how integration across five networks increases gradually with each task for the high bias group.

Post hoc analysis showed that significant changes occurred mainly between Task 1 and Task 2b across five out seven sparsity thresholds (T^0.20–0.40^: *t* = 2.429, *p* = 0.028; *t* = 2.43, *p* = 0.028; *t* = 2.335, *p* = 0.034; *t* = 2.318, *p* = 0.035; *t* = 2.345; *p* = 0.033). There was no significant difference in the prediction errors in Tasks 2a and 2b (Lim et al., 2020). Given that there was no change in task condition between these tasks, the lack of changes in SS indicated that prediction errors played a role only in changes observed between other scans. To test this assumption, the overall model was run without Task 2b and showed stronger effect (RM-ANOVA: *F*(12) = 5.563, *p* < 0.01 for type of scan × threshold). The between-subject effect was significant (*F*(1) = 4.739, *p* = 0.037).

### Relationships Between Segregation in Networks and Demographic Characteristics

To measure the effect of maladaptive characteristics related to pain, the relationships between SS and self-reported pain catastrophizing, anxiety, and depression measures were investigated.

Table 1 summarizes what demographic metrics were used and how they were associated with segregation in RSNs defined by different parcellations at the 0.20 sparsity threshold. Next, we report correlations that were significant at most sparsity thresholds only. Higher SS between five networks predicted higher magnification, an aspect of pain catastrophizing, at all seven sparsity thresholds (T^0.20–0.50^: *R* = 0.435, *p* = 0.006; *R* = 0.407, *p* = 0.01; *R* = 0.390, *p* = 0.014; *R* = 0.381, *p* = 0.017; *R* = 0.369, *p* = 0.021; *R* = 0.360, *p* = 0.024; *R* = 0.352, *p* = 0.028). In 17 networks, decreased integration was a significant indicator of state anxiety (T^0.20–0.50^: *R* = 0.371, *p* = 0.02; *R* = 0.396, *p* = 0.013; *R* = 0.388, *p* = 0.015; *R* = 0.392, *p* = 0.014; *R* = 0.384, *p* = 0.016; *R* = 0.377, *p* = 0.018; *R* = 0.369, *p* = 0.021) and trait anxiety (T^0.20–0.50^: *R* = 0.376, *p* = 0.018; *R* = 0.392, *p* = 0.014; *R* = 0.380, *p* = 0.017; *R* = 0.385, *p* = 0.015; *R* = 0.379, *p* = 0.017; *R* = 0.377, *p* = 0.018; *R* = 0.371, *p* = 0.02) at all seven sparsity thresholds.

**Table 1.** Relationship between system segregation and demographic characteristics for healthy controls (*n* = 39) at the 0.20 weighted threshold. Significant correlations are defined by asterisks (*). The symbol ^x^ indicates that nonparametric Spearman’s correlation analysis was performed on data instead of Pearson since data were not normally distributed. PCS = Pain Catastrophizing Scale.

Parameters	Mean ± *SD*	*R* value			*p* value		
Networks		5	7	17	5	7	17
Age^x^	31.41 ± 10.17	−0.291	−0.104	−0.251	0.073	0.527	0.123
PCS	12.44 ± 9.99	0.316*	0.261	0.219	0.050	0.109	0.180
Rumination^x^	5.54 ± 4.84	0.269	0.162	0.122	0.098	0.326	0.461
Magnification	2.82 ± 2.39	0.435*	0.196	0.166	0.006	0.231	0.313
Helplessness^x^	4.69 ± 4.68	0.177	0.291	0.243	0.281	0.072	0.136
State anxiety	32.18 ± 9.66	0.119	0.264	0.371*	0.505	0.104	0.020
Trait anxiety	35.80 ± 10.40	0.155	0.323*	0.376*	0.345	0.045	0.018

We assessed sex differences in top-down threat bias and weighted SS at different thresholds. There were no significant differences between male and female participants in threat bias (*p* = 0.574) and in SS (T^0.20–0.50^: *p* = 0.366; *p* = 0.254; *p* = 0.188; *p* = 0.151; *p* = 0.130; *p* = 0.111; *p* = 0.094). In addition, we confirmed that the group differences in SS were not related to head motion. We did not observe a significant difference in maximum FD and DVARS between the high and low bias groups for all the three network parcellations (*p* > 0.05). Similarly, there was no significant correlation between these two motion metrics and SS (*p* > 0.05).

## DISCUSSION

Here we report that higher segregation in RSNs was associated with greater amplification in pain during manipulated expectations of a stronger pain stimulus. Higher segregation in functional brain networks also associated with higher anxiety and pain catastrophizing. Our findings were reproducible at a wide range of network sparsity thresholds and between different types of predefined parcellations but were not significant when tested with subject-specific parcellations.

Both segregation and integration of brain networks have been recognized to be critical for cognitive functioning (Cohen & D’Esposito, 2016; Deco et al., 2015; Friston, 2009; Sporns, 2013; Wig, 2017). Previous work has shown that higher SS predicts different types of cognitive capacities and the ability to learn new physical and mental skills such as cognitive behavioral therapy and meditation (Arnemann et al., 2015; Baniqued et al., 2018, 2019; Cohen & D’Esposito, 2016; Gallen et al., 2016; Saghayi et al., 2020). More segregated networks also predicted higher expectation effects of placebo analgesia, suggesting the role of segregation in the process of integrating top-down information with sensory input (Hashmi et al., 2014). In the present study, we have found that higher segregation between canonical RSNs is associated with a top-down bias (or reduced capacity to integrate sensory evidence).

It has been suggested that individuals can vary in SS owing to underlying differences in neurotransmitter functions. A prominent hypothesis suggests that large-scale network dynamics are mediated by neuromodulatory neurotransmitter inputs from the ascending arousal system (Shine, 2019; Shine et al., 2016, 2018, 2019). Specifically, it has been shown that widespread projections from the noradrenergic locus coeruleus (LC) coordinate neural gain that in turn affects the balance between segregation and integration by altering network-level topology to promote attentional and cognitive performance (Shine et al., 2018; Wainstein et al., 2021). In addition, the patterns of segregation and integration are correlated with the spatial distribution of the *α*2a adrenergic receptors (Wainstein et al., 2021). We have previously demonstrated that *α*2 adrenergic receptors are important for maintaining brain network architecture by administering an agonist (dexmedetomidine) that significantly reduced resting-state functional connectivity both within and between brain subnetworks (Hashmi et al., 2017). The *α*2 adrenergic tone has a direct link with stress, stress resilience, and anxiety (Grueschow et al., 2021; McCall et al., 2015; Ross & Van Bockstaele, 2021). In rodents, stimulant drugs and physiological manipulations targeting these receptors can lead to heightened LC reactivity to stressors (Morris et al., 2020; Samuels & Szabadi, 2008). The heightened noradrenergic tone following an aversive threat can promote cued fear learning that in turn enhances amygdala function and impairs prefrontal structure and function (Giustino & Maren, 2018). This segues into the observed association between increased SS and higher state and trait anxiety and higher magnification, a subscale of pain catastrophizing. Previous studies have linked increased anxiety to decreased resting-state functional connectivity within/between the affective (AN), salience (SN), central executive (CEN), and default mode (DMN) networks (Xu et al., 2019). Excessive anxiety has been associated with lower functional connectivity between the DMN and the anterior SN (Gerlach et al., 2021). Since pain is an aversive signal, pain processes and aversive fear conditioning share similar pathways (Biggs et al., 2020; Ceko et al., 2022; Elman & Borsook, 2018; Ji et al., 2018). The observed association between SS, threat bias, and anxiety-prone behaviors taken together with what has been suggested about a role of adrenergic pathways in arousal and network integration suggests a role of altered autonomic nervous system function in fear of pain. In addition, the SS measure offers a global metric that can be investigated to study the putative link between adrenergic (and potentially other neurotransmitters) tone (Shine, 2019; Shine et al., 2018, 2019) and anxiety-prone patterns of responses to pain (Delgado-Gallén et al., 2023).

Previous work from our lab also indicates that white matter pathways that connect disparate brain regions show reduced integrity in people with high threat bias (Aristi et al., 2022). Specifically, people with high threat bias exhibit weaknesses in the splenium of the corpus collosum (Aristi et al., 2022). This region is a structural pathway that connects sensory regions with multimodal regions in parietal cortex and is important for integrating top-down information with bottom-up sensory input (Aristi et al., 2022; Fabri et al., 2001). We conclude that individual differences behind macroscale and microscale neurophysiological mechanisms account for variation in threat bias sensitivity and can be reflected in measures of large-scale functional dynamics such as SS.

We found that participants in the high bias group showed significant change in SS from rest to task while networks in individuals from the low bias group stayed consistently integrated across all scans. Several studies reported that large-scale brain networks stay less integrated during task performance compared with rest (Cohen & D’Esposito, 2016; Cole et al., 2014; Krienen et al., 2014; Shine et al., 2016). Whether the system is more integrated or segregated depends on task complexity that requires different amounts of cognitive control: simple tasks are associated with greater network segregation, while more complex tasks favor decreased segregation (Cohen & D’Esposito, 2016; Shine et al., 2016; Yue et al., 2017). These rest to task differences in segregation can be explained by the need to minimize metabolic cost of brain function (Bullmore & Sporns, 2012; Zalesky et al., 2014). Brain networks are wired to minimize metabolic costs by staying less clustered / more segregated at rest, but task-related processing demands engage communication and relocation of energy resources between specific task-relevant networks that become more connected/integrated (Bullmore & Sporns, 2012). Our results suggest that the high bias group required greater engagement of brain networks subserving greater cognitive efforts to integrate prior expectations and contradictory sensory stimuli and thus demonstrated higher variation in task-specific network organization across the scans.

Our results were produced based on two different cortical parcellations that involve known canonical RSNs. The seven and 17 networks in the Yeo parcellations are two subscale resolutions of brain regions that show high reproducibility of resting-state fMRI connectivity when assessed from a thousand subjects (Yeo et al., 2011). Parcellations derived from large groups of healthy individuals have provided important insights into study of brain topological organization and network properties (Buckner et al., 2013). These properties have allowed researchers to investigate brain behavioral associations with developmental, cognitive, and behavioral phenotypes. It has been shown that the size, location, and spatial arrangement, albeit consistent between subjects, does show individual-level variations (Braga & Buckner, 2017; Harrison et al., 2015; Laumann et al., 2015). Yet, it remains unclear whether topological metrics derived from subject-specific parcellations are comparable in their association with behavior relative to metrics from group-based parcellations (Hacker et al., 2013; Wig et al., 2014). Here, we tested the possibility of whether individual-specific network organization offers comparable information to group-averaged networks. We showed that segregation in the predefined five and seven RSNs was more robust in association with pain behavior. The higher resolution parcellations with 17 RSNs showed some correspondence but was less predictive relative to the spatially coarser resolution networks, and segregation in individual-specific RSNs obtained by Girvan-Newman and Louvain algorithms failed to predict threat bias. Overall, our results indicate that predefined group-averaged RSNs are better at capturing behavioral differences associated with threat bias in pain compared with subject-specific RSNs.

The main limitation of this study is the absence of a validation dataset to test the reproducibility of current findings. The small group sizes resulting from the K-means clustering algorithm may reduce the statistical power of group analyses. Nonetheless, our data provided sufficient power to establish neurobiological correlates of top-down threat bias in pain observable in resting-state fMRI. As we demonstrated, the choice of parcellation, spatial resolution of the parcellation, and type of scan (rest vs. task) leads to different results. Thus, a more thorough investigation of variables with a larger dataset and cognitive measurements can help to validate and extend the findings.

In summary, this study shows, for the first time, that segregation in canonical RSNs is a predictive of individual variance in threat bias. Specifically, we suggest that the balance between large-scale dynamics in RSNs is critical for the processes involved in integrating sensory information with top-down priors in response to threat. Increased SS is also related to maladaptive pain traits such as higher pain catastrophizing and increased pain responsiveness to threat. Therefore, higher integration may be an indicator of coordinated activity between multiple brain networks that allows proper sensory integration and reduced amplification of sensory responses to threats. Future work that focuses on further investigation of neurobiological mechanisms implicated in top-down cognitive biases in pain can benefit the development of interventions targeting chronic pain conditions.

## DATA AVAILABILITY

The data that support the findings of this study are available from the corresponding author upon request.

## SUPPORTING INFORMATION

Supporting information for this article is available at https://doi.org/10.1162/netn_a_00328.

## AUTHOR CONTRIBUTIONS

Veronika Pak: Formal analysis; Investigation; Methodology; Validation; Visualization; Writing – original draft; Writing – review & editing. Javeria Ali Hashmi: Conceptualization; Data curation; Formal analysis; Funding acquisition; Investigation; Methodology; Project administration; Resources; Software; Supervision; Validation; Visualization; Writing – review & editing.

## FUNDING INFORMATION

Javeria Ali Hashmi, Canadian Institute of Health Research. Javeria Ali Hashmi, NSERC Discovery Grant. Javeria Ali Hashmi, Nova Scotia Health Research Foundation (https://dx.doi.org/10.13039/501100000194). Javeria Ali Hashmi, Nova Scotia Health Authority (NSHA) Establishment Grant. Javeria Ali Hashmi, NSHA Fibromyalgia Research Grant. Javeria Ali Hashmi, Canada Research Chairs Program. Javeria Ali Hashmi, John R. Evans Leaders and Canada Innovation Funds (CFI-JELF).

## TECHNICAL TERMS

Top-down threat bias: Perceptual experience that shows amplification with an increase in expectations of strong noxious events.
System segregation: A measure of the strength of between-network brain connections relative to within-network brain connections.
Parcellation: Distinct partitions of the brain into smaller regions of interest (ROIs) and/or networks, based on specific characteristics such as structural or functional connectivity.
Sparsity threshold: An extent at which the network connections below a certain percentile or absolute value are removed.
