# Supplementary material for: Top-down threat bias in pain perception is predicted by higher segregation between resting-state networks
Source: Netw Neurosci. 2023 Dec 22;7(4):1248–65. doi: 10.1162/netn_a_00328 (PMC10631789; doi:10.1162/netn_a_00328)
Supplement: Supplementary file 1 [file netn-7-4-1248-s001.docx]

**Title:** Top-down threat bias in pain perception is predicted by higher segregation between resting-state networks

**Authors:** Veronika Pak^1,2^, Javeria Ali Hashmi^1,2^

^1^Department of Anesthesia, Pain Management, and Perioperative Medicine, Nova Scotia Health Authority, Halifax, NS, Canada

^2^Dalhousie University, Halifax, NS, Canada


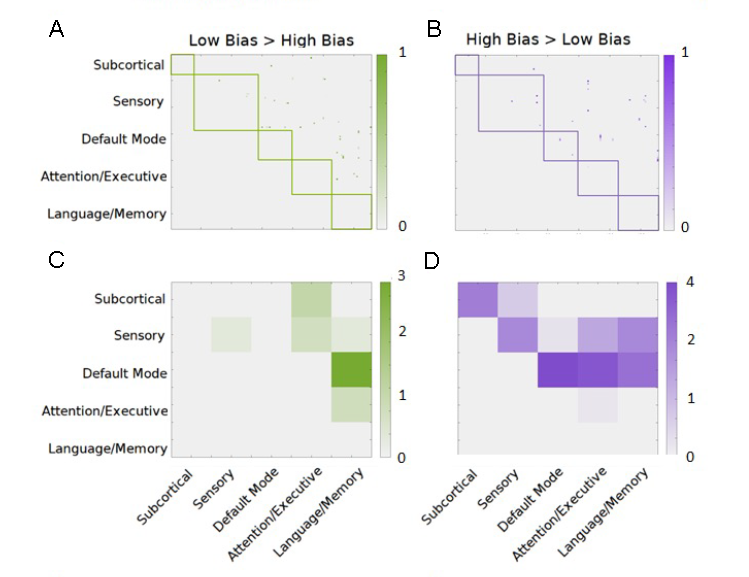


**Supplemental Figure 1 |** **A & B**: Contrast matrix showing all functional connections (edges) that were significantly higher in low bias (green) or high bias (purple), uncorrected. Nodes are ordered based on their affiliation with each of the five resting state networks, so that edges (green and purple pixels) can be visualized between specific nodes belonging to the five resting state networks. Green and purple margins show demarcations for the five resting state networks. The significant connections (edges p < 0.05) can be seen within (inside the margins) and between (outside the green margins) canonical resting state networks. **C & D**: Significant edges were summed from the contrast matrix (A & B) to depict the sum of corrected significant functional connections normalized by the number of all possible connections for the two contrasts, respectively. Colormap represents values of the normalized sum of significant connections. The high-bias group showed more significant connections within networks, while the low bias

group had more significant connections between networks.
